# Effects of *helicobacter pylori* on tumor microenvironment and immunotherapy responses

**DOI:** 10.3389/fimmu.2022.923477

**Published:** 2022-07-28

**Authors:** Ruiyi Deng, Huiling Zheng, Hongzhen Cai, Man Li, Yanyan Shi, Shigang Ding

**Affiliations:** ^1^ Peking University Third Hospital, Research Center of Clinical Epidemiology, Beijing, China; ^2^ Peking University Health Science Center, Peking University First Medical School, Beijing, China; ^3^ Peking University Third Hospital, Department of Gastroenterology, Beijing, China; ^4^ Peking University Health Science Center, Peking University Third Medical School, Beijing, China

**Keywords:** *Helicobacter pylori*, immune evasion, gastric cancer, microenvironment, immunotherapy

## Abstract

*Helicobacter pylori* is closely associated with gastric cancer. During persistent infection, *Helicobacter pylori* can form a microenvironment in gastric mucosa which facilitates the survival and colony formation of *Helicobacter pylori*. Tumor stromal cells are involved in this process, including tumor-associated macrophages, mesenchymal stem cells, cancer-associated fibroblasts, and myeloid-derived suppressor cells, and so on. The immune checkpoints are also regulated by *Helicobacter pylori* infection. *Helicobacter pylori* virulence factors can also act as immunogens or adjuvants to elicit or enhance immune responses, indicating their potential applications in vaccine development and tumor immunotherapy. This review highlights the effects of *Helicobacter pylori* on the immune microenvironment and its potential roles in tumor immunotherapy responses.

## Introduction

*Helicobacter pylori* is a gram-negative, helical, microaerophilic, and flagellated bacteria that colonizes the gastric mucosa in approximately 50% of the world population (1, 2). *Helicobacter pylori* infection is the main cause of gastric mucosal diseases such as gastric cancer (GC), chronic non-atrophic gastritis, atrophic gastritis, intestinal metaplasia, and dysplasia (3). GC is the fifth most common cancer and the fourth leading cause of cancer-related deaths worldwide (4). *H. pylori* is classified by the WHO as a class I carcinogen associated with the onset of GC, as chronic *H. pylori* infection leads to at least 75% of GC cases (5–8). 2% of *H. pylori* infected patients will develop GC (7).

Tumor growth is supported by oncogene-driven metabolic activities as well as by the microenvironment. Infection with *H. pylori* promotes gastric tumorigenesis, mainly by influencing the microenvironment (9). Virulence factors such as cytotoxin-associated gene A (CagA), vacuolating cytotoxin A (VacA), urease (Ure), arginase (Arg), lipopolysaccharide (LPS), and neutrophil-activating protein (NAP), enable *H. pylori* to survive and colonize the gastric mucosa, maintain chronic inflammation, and induce malignant changes within the gastric mucosa (1, 10–12). The immune system plays a pivotal role in eliminating *H. pylori* infection and controlling inflammation. Throughout a long-term co-existence with human hosts, *H. pylori* has developed several strategies to maintain a balance between the immune response and immune escape (13, 14). Through regulating tumor stromal cells, immune checkpoints, and other regulatory factors, *H. pylori* constructs a microenvironment that favors persistent colonization and facilitates tumorigenesis.

However, the influence of *H. pylori* on responses to immunotherapies and the prognosis of GC remains controversial (15–18). Recent studies have presented that *H. pylori* infection might affect the curative effect of tumor therapy by the induced immuno-regulation (19, 20). Besides, *H. pylori* virulence factors such as NAP, VacA, and Ure might elicit or enhance immune responses, which indicates the potential application in vaccine development and tumor immunotherapy (21, 22). These virulence factors are immunodominant antigens of *H. pylori* and might improve patient prognosis as immunogens or adjuvants in immunotherapy (23). Here, this review describes the mechanisms and effects of *H. pylori* on the immune microenvironment of GC and tumor immunotherapy responses.

## Effects of *H. pylori* on tumor stromal cells in gastric tumor immune microenvironment

**Figure 1:**
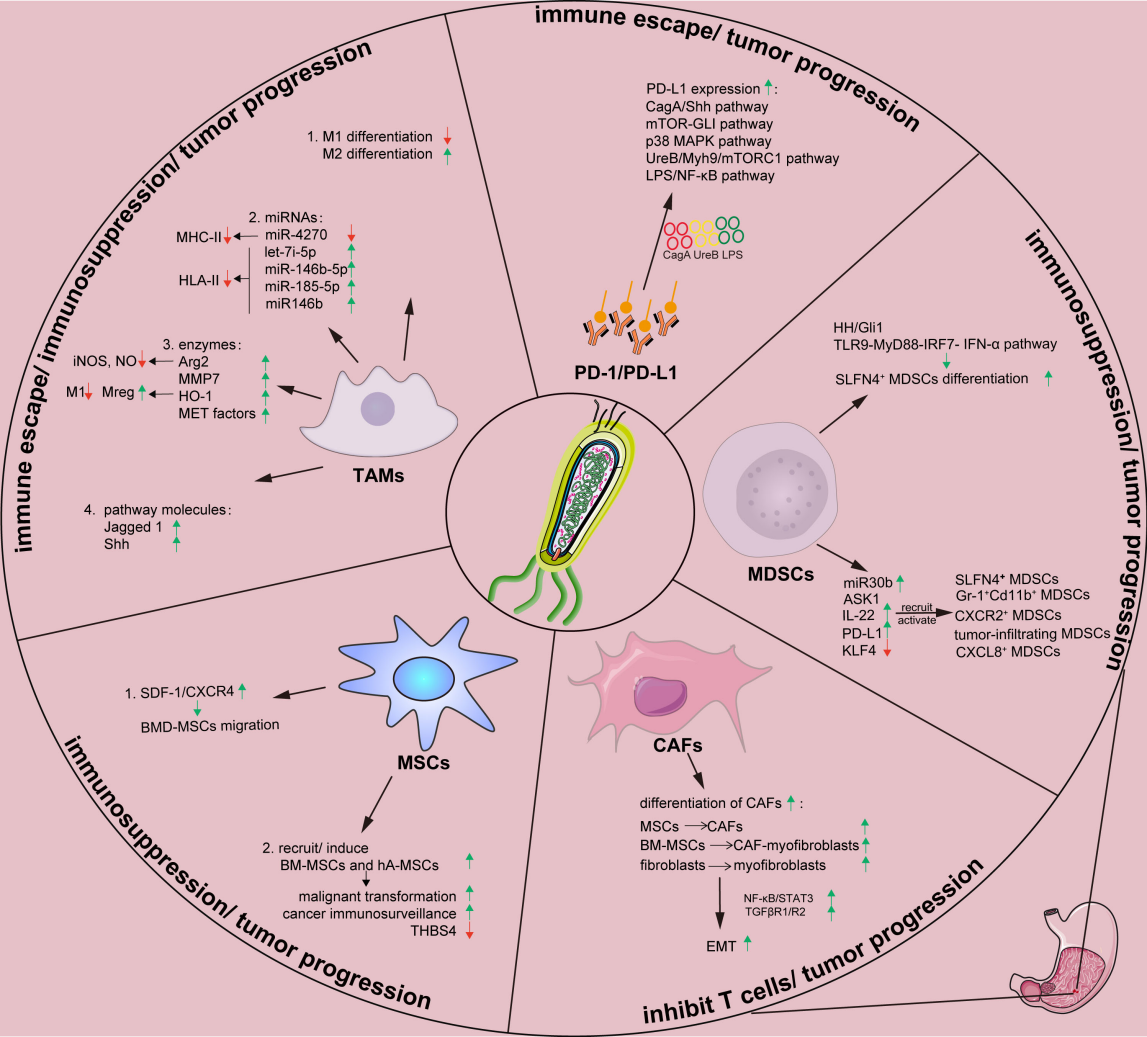
Effects of *H. pylori* on tumor stromal cells and tumor-related proteins in gastric tumor immune microenvironment. Arg, arginase; ASK1, apoptosis signal-regulating kinase 1; BM-MSC, Bone marrow-derived mesenchymal stem cells; CAF, cancer-associated fibroblast; Cag A, cytotoxin-associated gene A; CXCL8, chemokine (C-X-C motif) ligand 8; EMT, epithelial-mesenchymal transition; hA-MSC, human adipose-derived mesenchymal stem cells; HH, Hedgehog; HO-1, heme oxygenase-1; *H.pylori*, *Helicobacter pylori*; IL-22, Interleukin-22; IRF, interferon regulatory factor; IFN, interferon; KLF4, Krüppel-like factor 4; LPS, lipopolysaccharide; MAPK, mitogen-activated protein kinases; MDSCs, myeloid-derived suppressor cells; MET, mesenchymal-epithelial transition; MHC-II, major histocompatibility complex class II; MMP, matrix metalloproteinase; mTOR, mammalian target of rapamycin; Myh9, myosin heavy chain 9; NF-κB, nuclear factor kappa B; miR, microRNA; MSCs, mesenchymal stem cells; PD-1, programmed death 1; PD-L1, programmed death-ligand 1; PI3K-AKT, phosphatidylinositol 3 kinase-protein kinase B; ROS, reactive oxygen species; SDF, stromal-derived factor; Shh, Sonic hedgehog; SLFN4, Schlafen 4; STAT3, signal transducer and activator of transcription 3; TAMs, tumor-associated macrophages; TGFβ, transforming growth factor β; TLR, Toll-like receptor; Ure, urease; Vac A, vacuolating cytotoxin A.

The tumor microenvironment (TME) consists of a continuously evolving complex of tumor cells and stroma. Stroma comprises surrounding non-cancerous fibroblasts, epithelial, immune and blood cells, and extracellular components such as cytokines, growth factors, hormones, and extracellular matrix (ECM) (24, 25). Stroma plays a key role during tumor initiation, progression, and metastasis, meanwhile it significantly influences therapeutic responses and clinical outcomes (26). *Helicobacter pylori* and its virulence factors can form a microenvironment that facilitates its survival and colony formation by regulating the constituents and functions of the TME. This section summarizes the interactions between *H. pylori* and tumor stromal cells during GC initiation, progression, and metastasis and describes potential strategies to improve the prognosis (**Figure 1**; **Table 1**).

**Table 1:** Effects of *H. pylori* on tumor cells in gastric tumor immune microenvironment.

Tumor cells affected by *H. pylori*	Roles of *H. pylori*		Results
TAMs	Simultaneous impairment and induction of M1 macrophage and M2 macrophage differentiation, respectively, or transdifferentiation to M2 macrophages (27)		Promotes tumor progression and invasion by inducing angiogenesis and mediating immunosuppressive signals in solid tumors
	Regulation of specific miRNAs	Downregulates miR-4270 expression (28)	Impairs MHC-II expression and exposure, decreases antigen presentation ability, favors persistent *H. pylori* infection
		Upregulates let-7i-5p, miR-146b-5p, miR-185-5p, and miR146b expression (29, 30)	Inhibits HLA-II expression, compromises bacterial antigen presentation to Th lymphocytes, impairs immune responses to *H. pylori*
	Induces production of specific enzymes	Arg2 (31, 32)	Promotes immune escape of *H. pylori*, mediates macrophage apoptosis, restrains inflammatory responses
		MMP7 (33)	Promotes immune escape of *H. pylori*
		HO-1 (34)	Reduces M1 population, increases the number of Mregs, promotes immune escape of *H. pylori*
		MET factor (35)	Elicits uncontrolled activation of macrophages and inflammation involved in tumorigenesis and cancer development
	Regulation of some signaling pathway molecules	Upregulation of Jagged 1 expression (36)	Increases secretion of proinflammatory mediators and phagocytosis, decreases bacterial load, confers anti-bacterial activity on macrophages
		Induces SHH release from the stomach (37)	Induces macrophage migration during early *H. pylori* infection, involved in gastric immune response
MSCs	Upregulates CXCR4 expression and enhances MSCs migration toward SDF-1 (38)		Enhances BM-MSC migration into gastric tissues
	Recruits or induces BM-MSCs and hA-MSCs	Promotes malignant transformation (39–42)	Promotes *H. pylori*-mediated gastric tumorigenesis and development
		Mediates local and systemic immunosuppression (43, 44)	
		Alters THBS expression (45, 46)	
CAFs	Induces MSC differentiation into CAFs	Enhances expression of fibroblast markers, CAF activation, and levels of aggression/invasion markers (47, 48)	Promotes survival, proliferation, and migration of GC cell lines, inhibits antitumor functions of T cells in GC TME
	Stimulates BM-MSC differentiation into CAF myofibroblasts	Increases HDGF expression (49)	Enhances tumor cell ability to proliferate, invade, and metastasize (49, 50)
	Induces fibroblast transdifferentiation into myofibroblasts	Upregulates and downregulates HIF-1α and Bax expression, respectively (51)	Promotes gastric tumorigenesis
	Propels EMT *via* signal pathways and TGF‐β secretion	Induces activation or differentiation of rat gastric fibroblasts by NF-κB and STAT3 signaling (52)	Induces Snail1 expression and propels EMT leading to GC progression
		Secretes TGFβ1 and regulates TGFβR1/R2-dependent signaling in *H. pylori*-activated gastric fibroblasts (53–55)	Prompts reprogramming normal gastric epithelial cells towards a precancerous phenotype and promotes EMT in normal epithelial cells
MDSCs	Induces differentiation of SLFN4^+^ MDSCs	HH/Gli1 (56, 57)	Inhibits gastric inflammatory response by *H. pylor*i, suppresses T cell function, immune dysregulation, and tumor progression
		TLR9-MyD88-IRF7- IFN-α pathway (58)	
	Interaction between *H. pylori* and MDSCs is regulated by several factors	MiR130b (59)	Activates SLFN4^+^ MDSCs and promotes *H. pylori*-induced metaplasia
		ASK1 (25, 60)	Suppresses inflammation induced by infiltrating immature MDSCs
		IL-22 (61)	Induces expression of proinflammatory proteins, suppresses Th1 cell responses, promotes development of *H. pylori*-associated gastritis
		PD-L1 (62–64)	Promotes tumor infiltration of MDSCs, mediates resistance to anti-PD-1/PD-L1 therapy
		KLF-4 (65–67)	Promotes recruitment of MDSCs to tumors, creates immunosuppressive microenvironment, promotes tumor growth

### Effects of *H. pylori* on tumor-associated macrophages in gastric tumor immune microenvironment

Changes in immune responses and the immune escape of *H. pylori* are closely associated with tumor-associated macrophages (TAMs), which are emerging key players in the TME. Macrophages play crucial roles in host defense against bacterial infections and in the regulation of immune responses during *H. pylori* infection (68). However, macrophages can also induce angiogenesis and suppress the host immune response during cancer development (37, 69). Generally, TAMs comprise M1 and M2 subtypes (27). Proinflammatory activated M1 macrophages promote the type I T helper (Th1) immune response by producing type I proinflammatory cytokines such as IL-1β, IL-1α, and IL-6 to clear pathogens and inhibit tumor progression, while simultaneously suppressing Th2-type responses (27, 70, 71). Activated M2 macrophages contribute to production of ECM and anti-inflammatory effectors such as IL-4 and IL-10 that are involved in the Th2 immune response, promotion of wound healing, and suppression of Th1 responses (72–75). Additionally, a third type called regulatory macrophages (Mregs) secrete abundant IL-10 that limits inflammation but do not secrete ECM (72). *Helicobacter pylori* and other pathogens might impair M1 macrophage differentiation while inducing M2 macrophage differentiation or M1 transdifferentiation into M2 macrophages, which can promote tumor progression and invasion by inducing angiogenesis and mediating immunosuppressive signals in solid tumors (27).

Furthermore, *H. pylori* infection might regulate specific microRNAs (miRNAs) to control macrophage function and affect the TME (28, 76). Infection with *H. pylori* leads to the downregulated expression of miR-4270 by human monocyte-derived macrophages. This favors upregulation of expression of CD300E immune receptors that enhance the proinflammatory potential of macrophages. However, the expression and exposure of major histocompatibility complex class II (MHC-II) molecules on the plasma membrane are simultaneously compromised. Hence, antigen presentation ability is decreased, leading to persistent *H. pylori* infection (28). The upregulation of let-7i-5p, miR-146b-5p and miR-185-5p, and miR146b expression in macrophages caused by *H. pylori* infection can similarly decrease HLA-II expression on the plasma membrane, which ultimately compromises bacterial antigen presentation to Th lymphocytes and impairs immune responses against *H. pylori* (29, 30). Collectively, *H. pylori* infection mainly downregulates surface recognition factors at the transcriptional level by rendering macrophages fail to degrade the bacteria. Thus, macrophages become a protective niche for *H. pylori*.

*Helicobacter pylori* can induce the production of specific enzymes that regulate macrophage function and affect TME. The production of arginase II (Arg2) in macrophages induced by *H. pylori* infection results in cell apoptosis and restrained proinflammatory cytokine responses, thus promotes *H. pylori* immune evasion (31, 32). Matrix metalloproteinase 7 (MMP7) plays a pivotal role in *H. pylori*-mediated immune escape (33). Heme oxygenase-1 (HO-1) expression in macrophages also be induced, resulting in a polarization switch towards a reduction in the M1 population and an increase in the Mreg profile, causing innate and adaptive immune responses failure (34). Transfer exosomes expressing mesenchymal–epithelial transition (MET) factor, a cell-surface receptor tyrosine kinase from *H. pylori*‐infected GC cells, can elicit uncontrolled macrophage activation and downstream inflammation and might be associated with tumorigenesis and cancer development (35). These findings shed light on how *H. pylori* influences the gastric microenvironment by inducing the expression of macrophage-associated enzymes in TAMs.

Moreover, *H. pylori* upregulates the expression of Jagged 1, a ligand of Notch signaling that plays an important role in M1 macrophage activation and bactericidal activity to prevent *H. pylori* infection. Upregulated Jagged 1 expression induces an increase in the expression of proinflammatory mediators and phagocytosis and a decrease in the bacterial load, which together impart antibacterial activity in macrophages (36). The hedgehog (HH) signaling pathway also plays an important role in the gastric TME. Sonic hedgehog (SHH) induced by *H. pylori* infection acts as a macrophage chemoattractant, which is a prerequisite in the gastric immune response (37).

In conclusion, *H. pylori* infection at the early stage can induce the infiltration of polymorphonuclear leukocytes and mononuclear phagocytes in the gastric mucosa as an innate immune response (77). During the advanced stages of GC, *H. pylori* can escape immune surveillance by impairing the antigen presentation of TAMs or by disrupting the M1/M2 (or Mreg) balance in favor of an M2 (or Mreg) phenotype (34, 72). Immunosuppressive status eventually promotes tumorigenesis and cancer development (78). These mechanisms also provide the potential for investigating novel targeted drugs (79). Specific miRNAs such as let-7i-5p, miR-146b-5p, and miR-185-5p can be targeted to reduce adverse effects on macrophage antigen presentation (29). Targeting specific enzymes including MMP7 and HO-1 or signaling pathways, such as Notch and HH, to regulate the M1/M2 (or Mreg) balance might also warrant investigation (33, 34).

### Effects of *H. pylori* on recruiting and inducing bone marrow-derived mesenchymal stem cells in gastric tumor immune microenvironment

Multipotent mesenchymal stem cells (MSCs) can self-renew and differentiate into various cell types that play key roles in tissue healing, regeneration, and immune regulation (80). Bone marrow-derived mesenchymal stem cells (BM-MSCs) might play important roles in *H. pylori*-associated gastric tumorigenesis and immunosuppression. Upon sensing signals indicating gastric mucosa damage, BM-MSCs migrate from bone marrow to stomach *via* the peripheral circulation. BM-MSCs heal damaged mucosa through a paracrine mechanism and directed differentiation (81, 82). *H. pylori*-induced persistent inflammation is required for BM-MSC migration and tumorigenesis (43, 83). Upregulated C-X-C chemokine receptor type 4 (CXCR4) interacts with its ligand, stromal-derived factor (SDF-1) and then promote BM-MSC migration to the gastric tissues (38).

Gastric epithelial glands become repopulated with BM-MSCs in mice model one year after *H. pylori* infection (39). After recruitment to stomach, BM-MSCs can become entrapped in a microenvironment containing *H. pylori* and malignant cells, 25% of which originate from BM-MSCs. Fusion with epithelial cells might render BM-MSCs more susceptible to malignant transformation or lead to the promotion of cancerous processes (40). BM-MSCs gradually acquire a clonal advantage and undergo stepwise transformation to malignant cells (39). During malignant progression, gastric epithelial glandular units undergo monoclonal transformation, resulting in emerging cancer stem cell (CSC) clones and adenocarcinomas (39, 41). Human adipose-derived mesenchymal stem cells (hA-MSCs) also participate in gastric tumorigenesis by increasing tumor cells invasion and metastasis during *H. pylori* infection (42).

In addition to malignant transformation, MSCs can promote tumorigenesis locally and systemically by compromising cancer immune surveillance or altering tumor stroma. When transplanting BM-MSCs in *H. pylori* infected mice model, IL-10 and transforming growth factor-β1 (TGF-β1) can be increased, as well as T cells secreting IL-10 and CD4^+^ CD25^+^ Foxp3^+^ regulatory T (Treg) cells in splenic mononuclear cells (43, 44). BM-MSCs can reduce the fraction of T cells that produce IFN-γ, thus inhibiting CD4^+^ and CD8^+^ T cell proliferation. Local and systemic immunosuppression mediated by BM-MSCs contributes to GC development induced by *H. pylori* (43).

MSCs can also promote tumorigenesis by altering tumor stromal components. Thrombospondin (THBS) promotes tumorigenesis through crosstalk with BM-MSCs. Infection with *H. pylori* significantly upregulates the expression of THBS4 in BM-MSCs. Overexpressed THBS4 then mediates BM-MSC-induced angiogenesis in GC by activating the THBS4/integrin α2/PI3K/AKT pathway (45). Moreover, BM-MSCs can differentiate into pan-cytokeratin-positive (pan-CK^+^) epithelial cells and alpha-smooth muscle actin (α-SMA^+^) cancer-associated fibroblasts (CAFs) by secreting THBS2, thus promoting the development of *H. pylori*-associated GC (46).

BM-MSCs play pivotal roles in *H. pylori*-associated GC. The immune regulatory functions of MSCs remain obscure. Shedding light on these functions and their mechanisms will provide clues on therapeutic targets for preventing GC development.

### Effects of *H. pylori* on induction of cancer-associated fibroblasts in gastric tumor immune microenvironment

CAFs are activated myofibroblasts that accompany solid tumors and are principal constituents of tumor stroma (84, 85). They play important roles in the TME. They can create a niche for cancer cells and promote cancer progression by stimulating cancer cell proliferation, migration, invasion, and angiogenesis (85–87). Proinflammatory and tumor-associated factors secreted by CAFs might induce persistent inflammation or intervene in tumor immunity, thus mediate tumor immune escape (52, 88). Mainly derived from MSCs, CAFs could induce epithelial-mesenchymal transition (EMT), which enhances the invasive properties of malignant cells (89, 90) that detach from primary tumor site to surrounding tissues (91).

*Helicobacter pylori* infection can induce MSCs differentiating into CAFs, and upregulate the expression of fibroblast markers, fibroblast activation protein (FAP), CAF activation markers, and aggressive/invasive markers (47). FAP-positive CAFs enhance the survival, proliferation, and migration of GC cell lines and inhibit T cells function (48). *H. pylori* infection also increases the expression of hepatoma-derived growth factor (HDGF) (49, 50). Exposure to HDGF promotes the recruitment of BM-MSCs, stimulates their differentiation into CAF-myofibroblasts, and enhances tumor cell proliferation, invasiveness, and metastasis (49). Moreover, *H. pylori* infection can induce fibroblasts transdifferentiating into myofibroblasts, which upregulating the early carcinogenic marker hypoxia-inducible factor 1-alpha (HIF-1α) and downregulating proapoptotic bcl-2-like protein 4 (Bax) expression (51).

CAFs induced by *H. pylori* propel EMT by nuclear factor kappa B (NF-κB), signal transducer and activator of transcription 3 (STAT3), and TGF-β. *Helicobacter pylori* might induce the activation or differentiation of rat gastric fibroblasts *in vitro*, which then activate NF-κB and STAT3 signaling, and upregulate Snail1. This is an EMT-inducing transcription factor (EMT-TF) (52). As a major propeller of EMT in cancer progression and metastasis (53, 54), TGF-β can initiate tumorigenesis by activating EMT-type III initiation in epithelial cell compartments at the early stage of cancer development (55, 92). Gastric fibroblasts activated by *H. pylori* promote normal gastric epithelial cells to precancerous phenotype, and promote EMT by regulating TGFβ R1/R2-dependent signaling (55). The HH, Wnt, and Notch signaling pathways can interact with TGF-β pathway and induce EMT progression (93–97).

Collectively, persistent *H. pylori* infection increases the differentiation of CAFs, which propel EMT through NF-κB, STAT3, and TGF-β. As CAFs play key roles in the gastric microenvironment, targeting CAFs might be a potential strategy to improve the prognosis of patients (98, 99).

### Effects of *H. pylori* on myeloid-derived suppressor cells in gastric tumor immune microenvironment

Immature myeloid (progenitor) cells (IMCs) do not mediate immunosuppression in healthy individuals. However, chronic inflammation, infections, and autoimmune diseases impair IMC differentiation and decrease peripheral myeloid cells numbers, resulting in more myelopoiesis (100–103). This eventually results in myeloid-derived suppressor cells (MDSCs) accumulation and immunosuppression (102, 104). MDSCs mediate immune suppression by inducing immunosuppressive cells (105), blocking lymphocyte homing (106), producing reactive oxygen and nitrogen species (107, 108), exhausting critical metabolites for T cell function (109), expressing negative immune checkpoint molecules (110).

Interactions between *H. pylori* and MDSCs are important in gastric immune microenvironment. On one hand, *H. pylori* can induce the differentiation of myeloid cell differentiation factor Schlafen 4 (SLFN4^+^) MDSCs (56, 58). This factor marks a subset of MDSCs in the stomach during *H. pylori*-induced spasmolytic polypeptide-expressing metaplasia (SPEM) (57). During chronic *H. pylori* infection in mice model, a subset of HH-Gli1-dependent immune cells is recruited to the gastric epithelium, and polarizes into SLFN4^+^ MDSCs. Overexpression of the SHH ligand in infected WT mice accelerates SLFN4^+^ MDSCs differentiataion in gastric corpus (57). Furthermore, *H. pylori* can stimulate plasmacytoid dendritic cells to secrete IFN-α through toll-like receptor 9-myeloid differentiation factor 88-interferon regulatory factor 7 (TLR9-MyD88-IRF7 pathway) (58). Differentiated SLFN4^+^ MDSCs inhibit gastric inflammatory response induced by *H. pylori* and suppress T cell function (56–59). Persistent immune dysregulation then favors intestinal metaplasia and neoplastic transformation, which leads to immune disorders and tumor progression.

Several markers, such as MiR130b, apoptosis signal-regulating kinase 1 (ASK1), interleukin 22 (IL-22), programmed death-ligand 1 (PD-L1), and Krüppel-like factor 4 (KLF4) play regulatory roles in the interactions between *H. pylori* and MDSCs. MiR130b produced by SLFN4^+^ MDSCs suppress T cells function and promote *H. pylori*-induced metaplasia (59). ASK1 deficiency promotes a Th1-dependent immune response and recruits immature Gr-1^+^Cd11b^+^ MDSCs with *H. pylori* infection. This could lead to the development of gastric atrophy and metaplasia (25, 60). Moreover, IL-22 secreted by polarized Th22 cells induced by *H. pylori* can stimulate CXCL2 production from gastric epithelial cells. This causes CXCR2^+^ MDSCs migration to gastric mucosa, where they produce proinflammatory proteins and suppress Th1 cell responses, contributing to the development of *H. pylori*-associated gastritis (61). PD-L1 upregulation on the surface of gastric epithelial cells at the early stage of *H. pylori* infection (62) promotes tumor infiltration of MDSCs (63) and then lead to anti-PD-1/PD-L1 treatment resistance (64). KLF4 is an evolutionarily conserved zinc finger transcription factor and key regulator of diverse cellular processes (111–113). *Helicobacter pylori* and its virulence factor CagA can influence KLF4 expression. The transduction of CagA or infection with *H. pylori* downregulates KLF4 expression by inducing CXCL8 expression, and low KLF4 expression further upregulates CXCL8 expression (65). Increased CXCL8 expression promotes MDSCs recruitment to tumors as well as tumor growth, and creates an immunosuppressive microenvironment conducive to resistance against immune response (65–67).

A high abundance of MDSCs in patients correlate with more advanced GC and a poor prognosis (114, 115). MDSCs infiltration induced by *H. pylori* mediates immunosuppression, immune dysfunction, gastric tumorigenesis, and reduces the effect of chemotherapy and immunotherapy (63). The possibility that combining immunotherapy or chemotherapy with MDSC-targeting therapy might overcome drug resistance and improve prognosis warrants investigation (116–118).

## Effects of *H. pylori* on PD-1/PD-L1 in gastric tumor immune microenvironment

In addition to cells in TME, immune checkpoints are involved in regulating *H. pylori*-associated TME. (**Table 2**).

**Table 2:** Effects of *H. pylori* on tumor-related proteins in gastric tumor immune microenvironment.

Tumor-related proteins affected by *H. pylori*	Roles of *H. pylori*	Results
PD-1/PD-L1	Upregulates PD-1/PD-L1 expression (119–121)	Reduces excessive damage induced by *H. pylori*, reduces T cell-mediated cytotoxicity, promotes GC progression
	Upregulates PD-L1 expression by *H. pylori* CagA through the SHH pathway (62)	Inhibits T cell proliferation and Treg cell induction from naïve T cells, increases immune escape, promotes GC progression
	Upregulates PD-L1 expression by mTOR-GLI signaling (64)	
	Upregulates PD-L1 expression by the p38 MAPK pathway (122, 123)	
	Upregulates PD-L1 expression by *H. pylori* urease subunit through the Myh9/mTORC1 pathway (124)	
	Upregulates PD-L1 expression by *H. pylori* LPS through the NF-κB pathway (125)	

The 55 kDa transmembrane protein programmed death 1 (PD-1) is expressed in activated T cells, natural killer (NK) cells, B lymphocytes, macrophages, dendritic cells (DCs), and monocytes. It is abundantly expressed in tumor-specific T cells (126–128). PD-L1 (also known as CD274 or B7-H1) is a 33 kDa type 1 transmembrane glycoprotein that is widely expressed in macrophages, activated T lymphocytes, B cells, DCs, and also expressed in tumor cells (129). Binding of PD-1 and PD-L1 enhances T cell tolerance, inhibits T cell activation and proliferation, increases Th cell transformation to Foxp3^+^ Treg cell, and prevents T cell cytolysis in tumor cells (130). Thus, interaction between PD-1 and PD-L1 is a double-edged sword. It can inhibit immune responses and promote self-tolerance, while it can also lead to immune escape and tumor progression.

*Helicobacter pylori* infection could upregulate PD-1/PD-L1 expression in gastric ulcers and GC patients (119), which might be related with poor prognosis (131, 132). Chronic *H. pylori* infection could cause excessive damage to gastric mucosa. Upregulated PD-1/PD-L1 is launched to avoid such damage, meanwhile this also reduces T cell-mediated cytotoxicity and promotes GC progression (119–121). SHH pathway is involved in PD-L1 upregulating (62). As an HH transcriptional effector, zinc finger protein GL1, mediates mammalian target of rapamycin (mTOR)-induced PD-L1 expression in GC organoids (64). Kinds of *H. pylori* virulence factors are reported in this process. *H. pylori* T4SS components activate p38 MAPK pathway and upregulate PD-L1 expression, thus inhibiting T cell proliferation and inducing Treg differentiation from naïve T cells, which lead to immune escape (122, 123). *Helicobacter pylori* urease B subunit mediates PD-L1 upregulation *via* myosin heavy chain 9 (Myh9) or mTORC1 signaling in bone marrow-derived macrophages (BMDMs) and, and regulates CD8^+^ T cells infiltration and activation (124). *Helicobacter pylori* LPS induces PD-L1 expression *via* NF‐κB pathway in GC cells and eventually promotes GC progression (125).

Overall, PD-1/PD-L1 play vital roles in *H. pylori*-infected GC, which presents an opportunity and challenge for treatment. However, numerous unknown mechanisms of PD-1/PD-L1 expression might be the basis for overcoming drug resistance and developing novel immunotherapies (133). The mechanisms and functions of PD1/PD-L1 with *H. pylori* infection requires further investigation (132, 134–136).

## Effects of *H. pylori* on tumor immunotherapy responses

**Figure 2:**
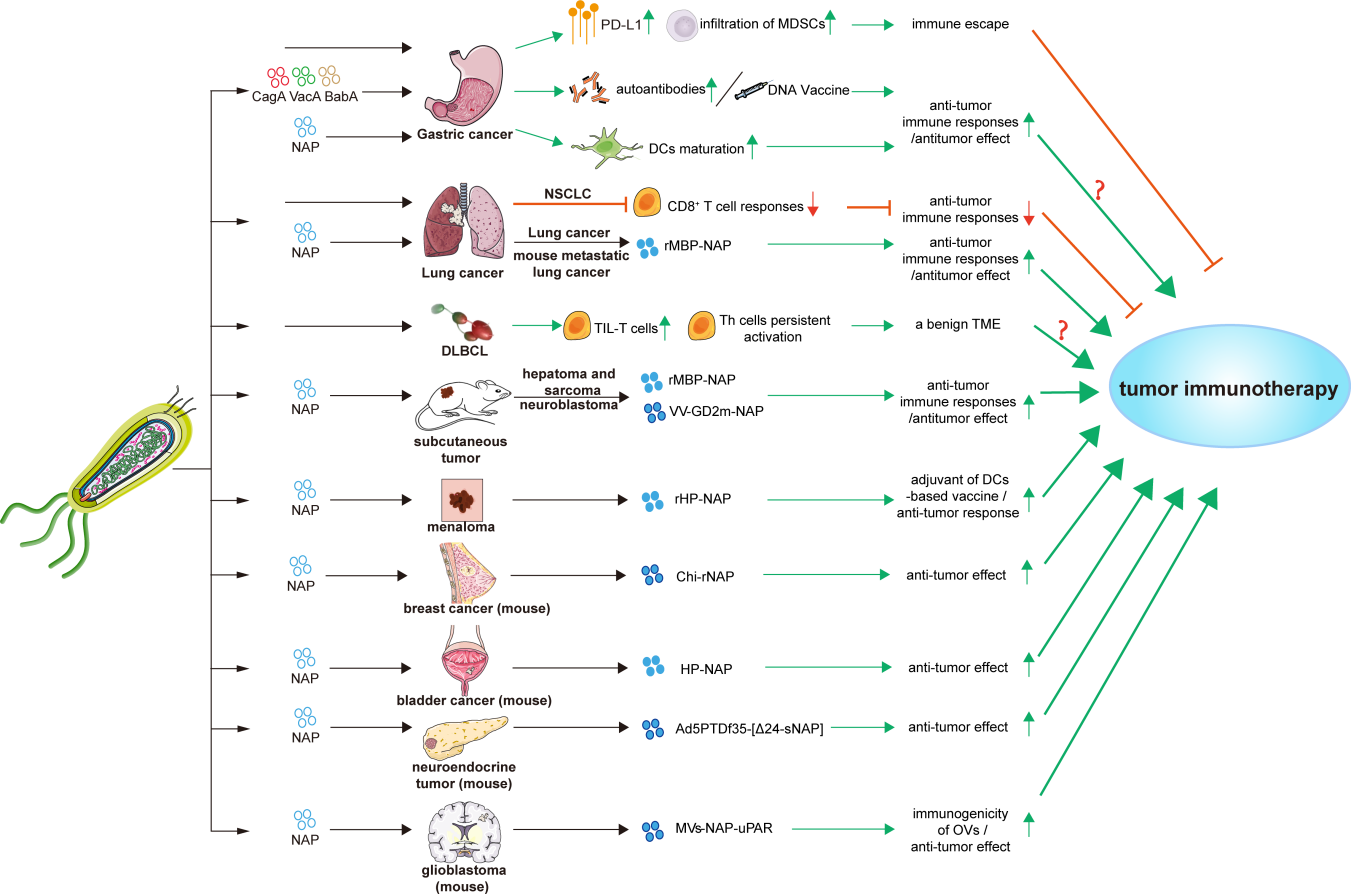
Effects and applications of *H. pylori* and its factors in tumor immunotherapies. Bab A, blood-group antigen-binding adhesin gene A; Cag A, cytotoxin-associated gene A; Chi-rNap, rNAP coated chitosan nanoparticles; DCs, dendritic cells; DLBCL, diffuse large B-cell lymphoma; HP-NAP, *H. pylori* neutrophil-activating protein; MDSCs, myeloid-derived suppressor cells; MV-NAP-uPAR, recombinant measles virus-NAP-urokinase-type plasminogen activator receptor; NSCLC, non-small cell lung cancer; OVs, oncolytic viruses; PD-L1, programmed death-ligand 1; rHP-NAP, recombinant *H. pylori* neutrophil-activating protein; rMBP-NAP, recombinant HP-NAP with the maltose-binding protein of Escherichia coli; Th cells, T helper cells; TIL-T cells, tumor-infiltrating T lymphocytes; TME, tumor microenvironment; Vac A, vacuolating cytotoxin A; VV-GD2m-NAP, vaccinia virus - neuroblastoma-associated antigen disialoganglioside mimotope.

Immunotherapy stimulates the immune system against neoplasms and harnesses the specificity of innate immune to fight cancer, particularly by activating T-cell mediated immunity (137, 138). With the wide application of immune therapy, the immune checkpoint inhibitors (ICIs) targeting immune checkpoint molecules such as PD-1 and CTLA-4, and other immune therapies such as cancer vaccine, the immune cells input, antigen vaccine, oncolytic viruses, and recombinant cytokines, have been receiving worldwide attention and have made a certain progress (139–147). However, as lack of optimal criteria selecting suitable patients until now, the objective response rate of immunotherapy remains low (148, 149). Hence, factors that influence the effectiveness of tumor immunotherapy need to be identified. In this section, we focused on the effects and potential applications of *H. pylori* infection on tumor immunotherapies (**Figure 2**; **Table 3**).

**Table 3:** Effects of *H. pylori* on tumor immunotherapy responses.

Cancer targeted by immunotherapy affected by *H. pylori*	Roles of *H. pylori*		Effects and applications
Gastric cancer	Induces PD-L1 expression and MDSC infiltration (62–64, 150)		Mediates immune escape by cancer cells, causing resistance to immunotherapy
	Enhances tumor immunity by virulence factors	CagA, VacA and BabA	Increases levels of CagA, VacA, and BabA autoantibodies, enhances antigen processing and presentation and T-cell activation and proliferation, and improves host immune status (151)
			DNA vaccine from CagA, VacA and BabA induces a shift from Th1 to Th2 response and activates CD3^+^ T cells to inhibit GC xenograft growth *in vivo* (152)
		HP-NAP	HP-NAP promotes maturation of DCs and stimulates neutrophils and monocytes to enhance antigen-specific T cell responses (153)
			Oral NapA vaccination promotes Th17 and Th1 polarization, exerts anti-*H. pylori* and antitumor effects, enhances immune responses (154)
Non-small cell lung carcinoma	Decreases immune responses, inhibits antitumoral CD8^+^ T cell responses (19)		Partially blocks the activity of ICIs and vaccine-based cancer immunotherapy
DLBCL	Causes increased numbers of tumor-infiltrating T lymphocytes and persistent activation of autoimmune Th cells (155)		Results in a benign tumor immune microenvironment
Mouse subcutaneous hepatoma and sarcoma	rMBP-NAP promotes Th1 differentiation and increases the number of CD4^+^ IFN-γ-secreting cells (156)		rMBP-NAP has antitumor potential
Lung cancer	rMBP-NAP increases the number of IFN−γ-secreting cells and CTL activity of PBMCs (157)		
Mouse metastatic lung cancer	rMBP-NAP restricts tumor progression by triggering antitumor immunity (158)		
Mouse breast and bladder cancers	HP-NAP enhances immune response and inhibits tumor growth (137, 159)		HP-NAP has antitumor potential
Melanoma	rHP-NAP promotes the maturation of dendritic cells in dendritic cell-based vaccines (160)		rHP-NAP has potential as an adjuvant
Mouse neuroendocrine tumor	HP-NAP improves median survival (161)		HP-NAP is a powerful source of immune-stimulatory agonists that can boost OV immunogenicity and enhance ICI effects (162, 163)
Mouse subcutaneous neuroblastoma	HP-NAP enhances antitumor efficacy of oncolytic vaccinia virus (164, 165)	
Glioblastoma	MVs-NAP-uPAR improves tumor immunotherapy efficacy (163)	

### Effects and applications of *H. pylori* and its factors on GC immunotherapy

The 5-year survival rate of advanced GC patients is <30%. Although platinum-fluoropyrimidine combination chemotherapy is the standard first-line treatment for advanced GC, its low complete response rate and severe adverse reactions have limited its application (63, 166). Novel effective therapies are urgently required. For example, PD-1 inhibitor pembrolizumab received accelerated approval from the US Food and Drug Administration (FDA) in 2017 to treat recurrent advanced or metastatic gastric or gastroesophageal junction adenocarcinomas expressing PD-L1 (63, 167–169).

*Helicobacter pylori* is a class I carcinogen associated with GC (170–172). The overall survival of GC diagnosis is reported to be higher for patients with *H. pylori* infection (17). *Helicobacter pylori* infection induces PD-L1 expression and MDSC infiltration that mediate immune escape. HH signaling activated by *H. pylori* infection induces PD-L1 expression and tumor cell proliferation in GC, resulting in cancer cell resistance to immunotherapy (150). In addition, *Helicobacter pylori* and its virulence factors can act as antigens or adjuvants to enhance tumor immunity.

*Helicobacter pylori* virulence factors, such as CagA, VacA, blood-group antigen-binding adhesin gene (BabA), and *H. pylori* neutrophil-activating protein (HP-NAP), can act as antigens or adjuvants to enhance tumor immunity. The stimulation of autoantibodies during antigen processing and presentation and subsequent T-cell activation and proliferation improves the host immune status, which can kill cancer cells and even suppress metastasis (151). Moreover, *H. pylori* DNA vaccines encoding fragments of CagA, VacA, and BabA can induce Th1 shift to Th2 response in immunized BALB/c mice, which mimics the immune status of GC patients with chronic *H. pylori* infection. Stimulated CD3^+^ T cells inhibit the proliferation of human GC cells *in vitro*, and the adoptive infusion of CD3^+^ T cells inhibits the growth of GC xenografts *in vivo* (152).

HP-NAP is a major virulence factor in *H. pylori* infection and colony formation, and it can also act as a protective factor (173, 174). As a Toll-like receptor-2 (TLR2) agonist, HP-NAP can bind to TLR2 of neutrophils (161, 175). Furthermore, HP-NAP promotes the maturation of DCs with Th1 polarization and improves migration of mature DCs. Stimulating neutrophils and monocytes by HP-NAP induces IL-12 and IL-23 expression, thus shifting antigen-specific T cell responses from the Th2 to the Th1 phenotype which characterized by abundant IFN-γ and TNF-α expression (153). Vaccination with HP-NAP A subunit (NapA) promotes Th17 and Th1 polarization. Such vaccines have potential effects as an anti-*H. pylori* oral vaccine candidate and a mucosal immunomodulatory agent, which could be used in antitumor strategies (154).

### Effects and applications of *H. pylori* and its factors in other tumor immunotherapies

In addition to GC, the influence of *H. pylori* on other tumor immunotherapies is also paid much attention recently. *Helicobacter pylori* infection might disrupt the immune system and exert detrimental effects on the outcomes of cancer immunotherapies (19).

*Helicobacter pylori* seropositivity could reduce anti-PD-1 immunotherapy effect in non-small cell lung cancer (NSCLC) patients. *Helicobacter pylori* infection partially blocks the activities of ICIs and vaccine-based cancer immunotherapies. *Helicobacter pylori* suppresses the innate and adaptive immune responses of infected hosts and inhibits antitumor CD8^+^ T cell responses by altering the cross-presentation activity of DCs (19). In contrast, a significantly high proportion of tumor-infiltrating T lymphocytes in *H. pylori*-positive *de novo* diffuse large B-cell lymphoma (DLBCL) patients preliminarily indicates a benign TME. Inflammation induced by *H. pylori* confers persistent activation of autoimmune Th cells, which would explain the benign TME (155). More researches are necessary to elucidate how *H. pylori* infection status influences the effects of tumor immunotherapies.

The immunomodulatory activity and potential applications of NAP in tumor immunotherapy have been investigated. Recombinant HP-NAP with the maltose-binding protein of *Escherichia coli* (rMBP-NAP) can mediate T helper lymphocytes differentiation into the Th1 phenotype and significantly increase the number of CD4^+^ IFN-γ-secreting T cells. This induces antitumor effects through a TLR-2-dependent mechanism in subcutaneous hepatoma and sarcoma mice model (156). rMBP-NAP can significantly increase peripheral blood mononuclear cells (PBMCs) that secrete IFN-γ, and prominently increases the cytotoxic activity of PBMCs derived from lung cancer patients (157). Treatment with rMBP-NAP restricts the progression of metastatic lung cancer in mice model by triggering antitumor immunity (158). A therapeutic nanocomplex of HP-NAP altered the production rate of cytokines and increase tumoricidal activities of the immune system, leading to decreased breast tumor growth in mice (137). Local administration of HP-NAP inhibits tumor growth by triggering tumor cell necrosis in bladder cancer mice model (159). Recombinant HP-NAP has potential effects as an adjuvant in DC-based vaccines for treating melanoma (160).

Because of its ideal immunogenicity, NAP has recently been applied as an immune adjuvant to enhance the antitumor immune response. When combined with oncolytic viruses (OVs), HP-NAP can activate the immune response. The intratumoral administration of adenovirus armed with secretory HP-NAP can improve the median survival rate of nude mice xenografted with neuroendocrine tumors (161). A recombinant vaccinia virus (VV) neuroblastoma-associated antigen disialoganglioside mimotope (GD2m)-NAP significantly improved therapeutic efficacy. *Helicobacter pylori*-NAP might help to overcome virus-mediated suppressive immune responses, resulting in improved anti-GD2 antibody production and a better therapeutic outcome (164, 165). Moreover, recombinant measles virus (MV)-NAP-urokinase-type plasminogen activator receptor (uPAR) can improve immunotherapeutic effects on glioblastoma with a better tumor prognosis and increased susceptibility to CD8^+^ T cell-mediated lysis. Overall, HP-NAP represents a potential immunostimulatory agonists which can boost the immunogenicity of OVs and enhance ICIs effects (162, 163).

In conclusion, *H. pylori* and its virulence factors could be closely related with personalized treatment strategies during tumor immunotherapies. The mechanisms of *H. pylori* infection in tumor immunotherapies requires further elucidation, and the translation of research findings to clinical applications should be accelerated.

## Summary

This review summarized current knowledge of the effects of *H. pylori* on the immune microenvironment of GC and tumor immunotherapy responses. *Helicobacter pylori* elicits powerful immune responses during surviving and colonizing gastric mucosa. *Helicobacter pylori* has also developed several strategies to evade recognition and disrupt immune function. The constituents and functions of stroma are regulated by *H. pylori* and its virulence factors to facilitate its survival and colony. Persistent *H. pylori* infection can induce immune evasion and tumorigenesis.

The stroma provides TME for tumor initiation and development after *H. pylori* persistent infection. Immunotherapy targeting tumor-associated immune cells is more mature and improved, particularly immunotherapy targeting T cells, such as ICIs. PD-1 inhibitor pembrolizumab has received approval from the US FDA in 2017 to treat recurrent advanced or metastatic gastric or gastroesophageal junction adenocarcinomas (167). While some clinical trials targeting non-immune cells in TME such as CAFs, MSCs, have failed to show promising efficacy in cancer patients (176–178). The main reason might be a lack of deep understanding of the fundamental mechanisms of stromal cells and elements as well as a lack of reliable biomarkers to guide stroma-targeted therapies (176). Of course, because of the important roles of regulating the immune response in TME, targeting TAMs is getting more and more attraction. For example, targeting colony-stimulating factor 1 receptor (CSF1R) signaling and the CCL2-CCR2 axis are developing drugs (179, 180). And there are some developing drugs to reprogram TAMs from a pro-tumor phenotype to an anti-tumor phenotype and interrupt the bad cycle between TAMs and tumor cells (176, 177), such as agonistic anti-CD40 antibodies (181), PI3Kγ inhibitors (182). These ongoing researches show good prospects in immunotherapy. Based on these, it seems that immunotherapy intervening tumor-associated immune cells may be more appropriate currently. However, we should also pay attention to the study of non-immune cells in TME. Further research on these cells may provide clues for developing new therapies in the future.

*H. pylori* infection might affect the tumor immunotherapy. Although *H. pylori* infection has been reported as a protective factor in GC immunotherapy while in NSCLC as a negative factor, the mechanisms and effect of *H. pylori* on GC immunotherapy still remains unclear (19, 183). *Helicobacter pylori* virulence factors can act as immunogens or adjuvants to elicit or enhance immune responses. Some *H. pylori* virulence factors such as HP-NAP, have been applied as adjuvants or combined with drugs in pan-tumor treatment to improve immunotherapeutic efficiency. The effects of *H. pylori* in TME should be further explored, and clinical applications should be performed to select the proper features of population for better immunotherapy benefits.

## Author contributions

RD and HZ searched the literature and wrote the manuscript. HC and ML re-checked the literature. YS and SD designed this study and revised the manuscript. All authors contributed to the article and approved the submitted version.

## Funding

This study was funded by the National Natural Science Foundation of China (Grant No. 81700496 and 81870386), Peking University Medicine Fund of Fostering Young Scholars’ Scientific and Technological Innovation (BMU2021PY002), and Key laboratory for Helicobacter pylori infection and upper gastrointestinal diseases, Beijing Key Laboratory (No.BZ0371).

## Conflict of interest

The authors declare that the research was conducted in the absence of any commercial or financial relationships that could be construed as a potential conflict of interest.

## Publisher’s note

All claims expressed in this article are solely those of the authors and do not necessarily represent those of their affiliated organizations, or those of the publisher, the editors and the reviewers. Any product that may be evaluated in this article, or claim that may be made by its manufacturer, is not guaranteed or endorsed by the publisher.

## References

[1] Baj J Forma A Sitarz M Portincasa P Garruti G Krasowska D. Helicobacter pylori virulence factors-mechanisms bacterial pathogenicity gastric microenvironment. Cells (2020) 10(1):27. doi: 10.3390/cells10010027 PMC7824444 33375694

[2] Mentis A Lehours P Megraud F. Epidemiology and diagnosis of helicobacter pylori infection. Helicobacter. (2015) 20(Suppl 1):1–7. doi: 10.1111/hel.12250 26372818

[3] Machlowska J Baj J Sitarz M Maciejewski R Sitarz R. Gastric cancer: Epidemiology, risk factors, classification, genomic characteristics and treatment strategies. Int J Mol Sci (2020) 21(11):4012. doi: 10.3390/ijms21114012 PMC7312039 32512697

[4] Sung H Ferlay J Siegel RL Laversanne M Soerjomataram I Jemal A. Global cancer statistics 2020: GLOBOCAN estimates of incidence and mortality worldwide for 36 cancers in 185 countries. CA Cancer J Clin (2021) 71(3):209–49. doi: 10.3322/caac.21660 33538338

[5] Plummer M Franceschi S Vignat J Forman D de Martel C. Global burden of gastric cancer attributable to helicobacter pylori. Int J Cancer (2015) 136(2):487–90. doi: 10.1002/ijc.28999 24889903

[6] McColl KE. Clinical practice. Helicobacter pylori Infect N Engl J Med (2010) 362(17):1597–604. doi: 10.1056/NEJMcp1001110 20427808

[7] Ishaq S Nunn L. Helicobacter pylori and gastric cancer: a state of the art review. Gastroenterol Hepatol Bed Bench. (2015) 8(Suppl 1):S6–S14. doi: 10.22037/ghfbb.v8iSupplement.653 26171139 PMC4495426

[8] Anwar W Armstrong BK Correa P Forman D Gentile JM Haswell-Elkins M. Schistosomes, liver flukes and helicobacter pylori. In: IARC working group on the evaluation of carcinogenic risks to humans, vol. 61. . Lyon: IARC Monogr Eval Carcinog Risks Hum. p. 1–241.

[9] Seeneevassen L Bessede E Megraud F Lehours P Dubus P Varon C. Gastric cancer: Advances in carcinogenesis research and new therapeutic strategies. Int J Mol Sci (2021) 22(7):3418. doi: 10.3390/ijms22073418 33810350 PMC8037554

[10] Liccardi G Pentimalli F. Cancer, immunity and inflammation. report from the CDD Cambridge conferences 2018 and 2019. Cell Death Dis (2019) 10(11):798. doi: 10.1038/s41419-019-2032-0 31641107 PMC6805861

[11] Grivennikov SI Greten FR Karin M. Immunity, inflammation, and cancer. Cell. (2010) 140(6):883–99. doi: 10.1016/j.cell.2010.01.025 PMC2866629 20303878

[12] Yolanda LV Sergio PD Hugo ES Isabel AF Rafael BZ Aldo TD. Gastric cancer progression associated with local humoral immune responses. BMC Cancer (2015) 15:924. doi: 10.1186/s12885-015-1858-9 26589831 PMC4654873

[13] Mejias-Luque R Gerhard M. Immune evasion strategies and persistence of helicobacter pylori. Curr Top Microbiol Immunol (2017) 400:53–71. doi: 10.1007/978-3-319-50520-6_3 28124149

[14] Song L Song M Rabkin CS Williams S Chung Y Van Duine J. Helicobacter pylori immunoproteomic profiles in gastric cancer. J Proteome Res (2021) 20(1):409–19. doi: 10.1021/acs.jproteome.0c00466 33108201

[15] Alexander SM Retnakumar RJ Chouhan D Devi TNB Dharmaseelan S Devadas K. Helicobacter pylori in human stomach: The inconsistencies in clinical outcomes and the probable causes. Front Microbiol (2021) 12:713955. doi: 10.3389/fmicb.2021.713955 34484153 PMC8416104

[16] Zhang MJ Chen DS Li S Chen L Qi YX Zhang CJ. Helicobacter pylori infection as a potential favorable factor for immune checkpoint inhibitor therapy for gastric cancer. Invest New Drugs (2021) 39(5):1436–8. doi: 10.1007/s10637-021-01122-5 33913072

[17] Fang X Liu K Cai J Luo F Yuan F Chen P. Positive helicobacter pylori status is associated with better overall survival for gastric cancer patients: evidence from case-cohort studies. Oncotarget. (2017) 8(45):79604–17. doi: 10.18632/oncotarget.18758 PMC5668073 29108340

[18] Li G Yu S Xu J Zhang X Ye J Wang Z. The prognostic role of helicobacter pylori in gastric cancer patients: A meta-analysis. Clin Res Hepatol Gastroenterol (2019) 43(2):216–24. doi: 10.1016/j.clinre.2018.08.012 30361060

[19] Oster P Vaillant L Riva E McMillan B Begka C Truntzer C. Helicobacter pylori infection has a detrimental impact on the efficacy of cancer immunotherapies. Gut. (2021) 71(3):457–66. doi: 10.1136/gutjnl-2020-323392 PMC8862014 34253574

[20] Shi Y Zheng H Wang M Ding S. Influence of helicobacter pylori infection on PD-1/PD-L1 blockade therapy needs more attention. Helicobacter. (2022) 27(2):e12878. doi: 10.1111/hel.12878 35112435

[21] Mohammadzadeh R Soleimanpour S Pishdadian A Farsiani H. Designing and development of epitope-based vaccines against helicobacter pylori. Crit Rev Microbiol (2021), 1–24. doi: 10.1080/1040841X.2021.1979934 34559599

[22] Del Giudice G Malfertheiner P Rappuoli R. Development of vaccines against helicobacter pylori. Expert Rev Vaccines (2009) 8(8):1037–49. doi: 10.1586/erv.09.62 19627186

[23] Fu HW. Helicobacter pylori neutrophil-activating protein: from molecular pathogenesis to clinical applications. World J Gastroenterol (2014) 20(18):5294–301. doi: 10.3748/wjg.v20.i18.5294 PMC4017044 24833859

[24] Hinshaw DC Shevde LA. The tumor microenvironment innately modulates cancer progression. Cancer Res (2019) 79(18):4557–66. doi: 10.1158/0008-5472.CAN-18-3962 PMC6744958 31350295

[25] Navashenaq JG Shabgah AG Banach M Jamialahmadi T Penson PE Johnston TP. The interaction of helicobacter pylori with cancer immunomodulatory stromal cells: New insight into gastric cancer pathogenesis. Semin Cancer Biol (2021) S1044-579X(21):00248–0. doi: 10.1016/j.semcancer.2021.09.014 34600095

[26] Wu T Dai Y. Tumor microenvironment and therapeutic response. Cancer Lett (2017) 387:61–8. doi: 10.1016/j.canlet.2016.01.043 26845449

[27] Gambardella V Castillo J Tarazona N Gimeno-Valiente F Martinez-Ciarpaglini C Cabeza-Segura M. The role of tumor-associated macrophages in gastric cancer development and their potential as a therapeutic target. Cancer Treat Rev (2020) 86:102015. doi: 10.1016/j.ctrv.2020.102015 32248000

[28] Pagliari M Munari F Toffoletto M Lonardi S Chemello F Codolo G. Helicobacter pylori affects the antigen presentation activity of macrophages modulating the expression of the immune receptor CD300E through miR-4270. Front Immunol (2017) 8:1288. doi: 10.3389/fimmu.2017.01288 29085364 PMC5649134

[29] Codolo G Toffoletto M Chemello F Coletta S Soler Teixidor G Battaggia G. Helicobacter pylori dampens HLA-II expression on macrophages *via* the up-regulation of miRNAs targeting CIITA. Front Immunol (2019) 10:2923. doi: 10.3389/fimmu.2019.02923 31969878 PMC6960189

[30] Coletta S Battaggia G Della Bella C Furlani M Hauke M Faass L. ADP-heptose enables helicobacter pylori to exploit macrophages as a survival niche by suppressing antigen-presenting HLA-II expression. FEBS Lett (2021) 595(16):2160–8. doi: 10.1002/1873-3468.14156 34216493

[31] Lewis ND Asim M Barry DP de Sablet T Singh K Piazuelo MB. Immune evasion by helicobacter pylori is mediated by induction of macrophage arginase II. J Immunol (2011) 186(6):3632–41. doi: 10.4049/jimmunol.1003431 PMC3069806 21296975

[32] Hardbower DM Asim M Murray-Stewart T Casero RA Jr. Verriere T Lewis ND. Arginase 2 deletion leads to enhanced M1 macrophage activation and upregulated polyamine metabolism in response to helicobacter pylori infection. Amino Acids (2016) 48(10):2375–88. doi: 10.1007/s00726-016-2231-2 PMC5042810 27074721

[33] Krakowiak MS Noto JM Piazuelo MB Hardbower DM Romero-Gallo J Delgado A. Matrix metalloproteinase 7 restrains helicobacter pylori-induced gastric inflammation and premalignant lesions in the stomach by altering macrophage polarization. Oncogene. (2015) 34(14):1865–71. doi: 10.1038/onc.2014.135 PMC4237684 24837365

[34] Gobert AP Verriere T Asim M Barry DP Piazuelo MB de Sablet T. Heme oxygenase-1 dysregulates macrophage polarization and the immune response to helicobacter pylori. J Immunol (2014) 193(6):3013–22. doi: 10.4049/jimmunol.1401075 PMC4171064 25108023

[35] Che Y Geng B Xu Y Miao X Chen L Mu X. Helicobacter pylori-induced exosomal MET educates tumour-associated macrophages to promote gastric cancer progression. J Cell Mol Med (2018) 22(11):5708–19. doi: 10.1111/jcmm.13847 PMC6201349 30160350

[36] Wen J Chen C Luo M Liu X Guo J Wei T. Notch signaling ligand Jagged1 enhances macrophage-mediated response to helicobacter pylori. Front Microbiol (2021) 12:692832. doi: 10.3389/fmicb.2021.692832 34305857 PMC8297740

[37] Schumacher MA Donnelly JM Engevik AC Xiao C Yang L Kenny S. Gastric sonic hedgehog acts as a macrophage chemoattractant during the immune response to helicobacter pylori. Gastroenterology. (2012) 142(5):1150–9.e6. doi: 10.1053/j.gastro.2012.01.029 22285806 PMC3335966

[38] Fakhari S Kalantar E Nikzaban M Hakhamneshi MS Fathi F Nikkhoo B. Effect of helicobacter pylori infection on stromal-derived factor-1/CXCR4 axis in bone marrow-derived mesenchymal stem cells. Adv BioMed Res (2014) 3:19. doi: 10.4103/2277-9175.124650 24592369 PMC3929140

[39] Varon C Dubus P Mazurier F Asencio C Chambonnier L Ferrand J. Helicobacter pylori infection recruits bone marrow-derived cells that participate in gastric preneoplasia in mice. Gastroenterology. (2012) 142(2):281–91. doi: 10.1053/j.gastro.2011.10.036 22062361

[40] Ferrand J Lehours P Schmid-Alliana A Megraud F Varon C. Helicobacter pylori infection of gastrointestinal epithelial cells *in vitro* induces mesenchymal stem cell migration through an NF-kappaB-dependent pathway. PLoS One (2011) 6(12):e29007. doi: 10.1371/journal.pone.0029007 22216156 PMC3247220

[41] Alison MR Islam S Wright NA. Stem cells in cancer: instigators and propagators? J Cell Sci (2010) 123(Pt 14):2357–68. doi: 10.1242/jcs.054296 20592182

[42] Moradi SL Eslami G Goudarzi H Hajishafieeha Z Soleimani M Mohammadzadeh A. Role of helicobacter pylori on cancer of human adipose-derived mesenchymal stem cells and metastasis of tumor cells-an *in vitro* study. Tumou Biol (2016) 37(3):3371–8. doi: 10.1007/s13277-015-4137-0 26446460

[43] Lin R Ma H Ding Z Shi W Qian W Song J. Bone marrow-derived mesenchymal stem cells favor the immunosuppressive T cells skewing in a helicobacter pylori model of gastric cancer. Stem Cells Dev (2013) 22(21):2836–48. doi: 10.1089/scd.2013.0166 23777268

[44] Yaghoobi M. Bone marrow-derived stem cells in pathogenesis of helicobacter pylori-associated gastric cancer. Clin Transl Gastroenterol (2015) 6:e110. doi: 10.1038/ctg.2015.35 26355434 PMC5176315

[45] He L Wang W Shi H Jiang C Yao H Zhang Y. THBS4/integrin alpha2 axis mediates BM-MSCs to promote angiogenesis in gastric cancer associated with chronic helicobacter pylori infection. Aging (Albany NY) (2021) 13(15):19375–96. doi: 10.18632/aging.203334 PMC8386559 34390328

[46] Shi H Qi C Meng L Yao H Jiang C Fan M. Bone marrow-derived mesenchymal stem cells promote helicobacter pylori-associated gastric cancer progression by secreting thrombospondin-2. Cell Prolif (2021) 54(10):e13114. doi: 10.1111/cpr.13114 34435402 PMC8488559

[47] Zhang Q Wang M Huang F Yang T Cai J Zhang X. H. pylori infection-induced MSC differentiation into CAFs promotes epithelial-mesenchymal transition in gastric epithelial cells. Int J Mol Med (2013) 32(6):1465–73. doi: 10.3892/ijmm.2013.1532 24145921

[48] Wen X He X Jiao F Wang C Sun Y Ren X. Fibroblast activation protein-alpha-Positive fibroblasts promote gastric cancer progression and resistance to immune checkpoint blockade. Oncol Res (2017) 25(4):629–40. doi: 10.3727/096504016X14768383625385 PMC7841289 27983931

[49] Liu CJ Wang YK Kuo FC Hsu WH Yu FJ Hsieh S. Helicobacter pylori infection-induced hepatoma-derived growth factor regulates the differentiation of human mesenchymal stem cells to myofibroblast-like cells. Cancers (Basel) (2018) 10(12):479. doi: 10.3390/cancers10120479 PMC6316704 30513684

[50] Lee KH Choi EY Kim MK Lee SH Jang BI Kim TN. Hepatoma-derived growth factor regulates the bad-mediated apoptotic pathway and induction of vascular endothelial growth factor in stomach cancer cells. Oncol Res (2010) 19(2):67–76. doi: 10.3727/096504010X12864748215043 21302807

[51] Krzysiek-Maczka G Targosz A Ptak-Belowska A Korbut E Szczyrk U Strzalka M. Molecular alterations in fibroblasts exposed to helicobacter pylori: a missing link in bacterial inflammation progressing into gastric carcinogenesis? J Physiol Pharmacol (2013) 64(1):77–87. 23568974

[52] Krzysiek-Maczka G Targosz A Szczyrk U Strzalka M Brzozowski T Ptak-Belowska A. Involvement of epithelial-mesenchymal transition-inducing transcription factors in the mechanism of helicobacter pylori-induced fibroblasts activation. J Physiol Pharmacol (2019) 70(5):727–36. doi: 10.26402/jpp.2019.5.08 31889044

[53] Krzysiek-Maczka G Wrobel T Targosz A Szczyrk U Strzalka M Ptak-Belowska A. Helicobacter pylori-activated gastric fibroblasts induce epithelial-mesenchymal transition of gastric epithelial cells *in vitro* in a TGF-beta-dependent manner. Helicobacter. (2019) 24(5):e12653. doi: 10.1111/hel.12653 31411795

[54] Katsuno Y Lamouille S Derynck R. TGF-beta signaling and epithelial-mesenchymal transition in cancer progression. Curr Opin Oncol (2013) 25(1):76–84. doi: 10.1097/CCO.0b013e32835b6371 23197193

[55] Krzysiek-Maczka G Targosz A Szczyrk U Wrobel T Strzalka M Brzozowski T. Long-term helicobacter pylori Infect switches gastric epithelium reprogram towards cancer stem cell-related differ program Hp-activated gastric fibroblast-TGFbeta dependent manner. Microorganisms (2020) 8(10):1519. doi: 10.3390/microorganisms8101519 PMC7599721 33023180

[56] El-Zaatari M Kao JY Tessier A Bai L Hayes MM Fontaine C. Gli1 deletion prevents helicobacter-induced gastric metaplasia and expansion of myeloid cell subsets. PLoS One (2013) 8(3):e58935. doi: 10.1371/journal.pone.0058935 23520544 PMC3592845

[57] Ding L Hayes MM Photenhauer A Eaton KA Li Q Ocadiz-Ruiz R. Schlafen 4-expressing myeloid-derived suppressor cells are induced during murine gastric metaplasia. J Clin Invest (2016) 126(8):2867–80. doi: 10.1172/JCI82529 PMC4966326 27427984

[58] Xiang X Wu Y Li H Li C Yan L Li Q. Plasmacytoid dendritic cell-derived type I interferon is involved in helicobacter pylori infection-induced differentiation of schlafen 4-expressing myeloid-derived suppressor cells. Infect Immun (2021) 89(11):e0040721. doi: 10.1128/IAI.00407-21 34370509 PMC8519294

[59] Ding L Li Q Chakrabarti J Munoz A Faure-Kumar E Ocadiz-Ruiz R. MiR130b from Schlafen4(+) MDSCs stimulates epithelial proliferation and correlates with preneoplastic changes prior to gastric cancer. Gut. (2020) 69(10):1750–61. doi: 10.1136/gutjnl-2019-318817 PMC7377952 31980446

[60] Hayakawa Y Hirata Y Hata M Tsuboi M Oya Y Kurokawa K. Dysregulated immune responses by ASK1 deficiency alter epithelial progenitor cell fate and accelerate metaplasia development during h. pylori infection. Microorganisms (2020) 8(12):1995. doi: 10.3390/microorganisms8121995 33542169 PMC7765114

[61] Zhuang Y Cheng P Liu XF Peng LS Li BS Wang TT. A pro-inflammatory role for Th22 cells in helicobacter pylori-associated gastritis. Gut. (2015) 64(9):1368–78. doi: 10.1136/gutjnl-2014-307020 PMC4552937 25134787

[62] Holokai L Chakrabarti J Broda T Chang J Hawkins JA Sundaram N. Increased programmed death-ligand 1 is an early epithelial cell response to helicobacter pylori infection. PLoS Pathog (2019) 15(1):e1007468. doi: 10.1371/journal.ppat.1007468 30703170 PMC6380601

[63] Kim W Chu TH Nienhuser H Jiang Z Del Portillo A Remotti HE. PD-1 signaling promotes tumor-infiltrating myeloid-derived suppressor cells and gastric tumorigenesis in mice. Gastroenterology. (2021) 160(3):781–96. doi: 10.1053/j.gastro.2020.10.036 PMC7878361 33129844

[64] Koh V Chakrabarti J Torvund M Steele N Hawkins JA Ito Y. Hedgehog transcriptional effector GLI mediates mTOR-induced PD-L1 expression in gastric cancer organoids. Cancer Lett (2021) 518:59–71. doi: 10.1016/j.canlet.2021.06.007 34126195 PMC8606306

[65] Liu Z Wu X Tian Y Zhang W Qiao S Xu W. H. pylori infection induces CXCL8 expression and promotes gastric cancer progress through downregulating KLF4. Mol Carcinog (2021) 60(8):524–37. doi: 10.1002/mc.23309 34038586

[66] Li BH Garstka MA Li ZF. Chemokines and their receptors promoting the recruitment of myeloid-derived suppressor cells into the tumor. Mol Immunol (2020) 117:201–15. doi: 10.1016/j.molimm.2019.11.014 31835202

[67] Alfaro C Teijeira A Onate C Perez G Sanmamed MF Andueza MP. Tumor-produced interleukin-8 attracts human myeloid-derived suppressor cells and elicits extrusion of neutrophil extracellular traps (NETs). Clin Cancer Res (2016) 22(15):3924–36. doi: 10.1158/1078-0432.CCR-15-2463 26957562

[68] Zhang X Arnold IC Muller A. Mechanisms of persistence, innate immune activation and immunomodulation by the gastric pathogen helicobacter pylori. Curr Opin Microbiol (2020) 54:1–10. doi: 10.1016/j.mib.2020.01.003 32007716

[69] Kaparakis M Walduck AK Price JD Pedersen JS van Rooijen N Pearse MJ. Macrophages are mediators of gastritis in acute helicobacter pylori infection in C57BL/6 mice. Infect Immun (2008) 76(5):2235–9. doi: 10.1128/IAI.01481-07 PMC2346689 18332213

[70] Mosser DM Edwards JP. Exploring the full spectrum of macrophage activation. Nat Rev Immunol (2008) 8(12):958–69. doi: 10.1038/nri2448 PMC2724991 19029990

[71] Wynn TA. Type 2 cytokines: mechanisms and therapeutic strategies. Nat Rev Immunol (2015) 15(5):271–82. doi: 10.1038/nri3831 25882242

[72] Cassetta L Cassol E Poli G. Macrophage polarization in health and disease. ScientificWorldJournal. (2011) 11:2391–402. doi: 10.1100/2011/213962 PMC3236674 22194670

[73] Biswas SK Chittezhath M Shalova IN Lim JY. Macrophage polarization and plasticity in health and disease. Immunol Res (2012) 53(1-3):11–24. doi: 10.1007/s12026-012-8291-9 22418728

[74] Qian BZ Pollard JW. Macrophage diversity enhances tumor progression and metastasis. Cell. (2010) 141(1):39–51. doi: 10.1016/j.cell.2010.03.014 20371344 PMC4994190

[75] Gordon S. Alternative activation of macrophages. Nat Rev Immunol (2003) 3(1):23–35. doi: 10.1038/nri978 12511873

[76] Fabian MR Sonenberg N. The mechanics of miRNA-mediated gene silencing: a look under the hood of miRISC. Nat Struct Mol Biol (2012) 19(6):586–93. doi: 10.1038/nsmb.2296 22664986

[77] Wilson KT Crabtree JE. Immunology of helicobacter pylori: insights into the failure of the immune response and perspectives on vaccine studies. Gastroenterology. (2007) 133(1):288–308. doi: 10.1053/j.gastro.2007.05.008 17631150

[78] Huber V Camisaschi C Berzi A Ferro S Lugini L Triulzi T. Cancer acidity: An ultimate frontier of tumor immune escape and a novel target of immunomodulation. Semin Cancer Biol (2017) 43:74–89. doi: 10.1016/j.semcancer.2017.03.001 28267587

[79] Pulendran B Davis MM. The science and medicine of human immunology. Science. (2020) 369(6511):eaay4014. doi: 10.1126/science.aay4014 32973003 PMC7872131

[80] Baj J Korona-Glowniak I Forma A Maani A Sitarz E Rahnama-Hezavah M. Mechanisms of the epithelial-mesenchymal transition and tumor microenvironment in helicobacter pylori-induced gastric cancer. Cells (2020) 9(4):1055. doi: 10.3390/cells9041055 PMC7225971 32340207

[81] Fu X Liu G Halim A Ju Y Luo Q Song AG. Mesenchymal stem cell migration and tissue repair. Cells. (2019) 8(8):784. doi: 10.3390/cells8080784 PMC6721499 31357692

[82] Baker N Boyette LB Tuan RS. Characterization of bone marrow-derived mesenchymal stem cells in aging. Bone. (2015) 70:37–47. doi: 10.1016/j.bone.2014.10.014 25445445

[83] Houghton J Stoicov C Nomura S Rogers AB Carlson J Li H. Gastric cancer originating from bone marrow-derived cells. Science. (2004) 306(5701):1568–71. doi: 10.1126/science.1099513 15567866

[84] Arcucci A Ruocco MR Granato G Sacco AM Montagnani S. Cancer: An oxidative crosstalk between solid tumor cells and cancer associated fibroblasts. BioMed Res Int (2016) 2016:4502846. doi: 10.1155/2016/4502846 27595103 PMC4993917

[85] Karagiannis GS Poutahidis T Erdman SE Kirsch R Riddell RH Diamandis EP. Cancer-associated fibroblasts drive the progression of metastasis through both paracrine and mechanical pressure on cancer tissue. Mol Cancer Res (2012) 10(11):1403–18. doi: 10.1158/1541-7786.MCR-12-0307 PMC4399759 23024188

[86] Yamaguchi H Sakai R. Direct interaction between carcinoma cells and cancer associated fibroblasts for the regulation of cancer invasion. Cancers (Basel) (2015) 7(4):2054–62. doi: 10.3390/cancers7040876 PMC4695876 26473929

[87] Kalluri R Zeisberg M. Fibroblasts in cancer. Nat Rev Cancer (2006) 6(5):392–401. doi: 10.1038/nrc1877 16572188

[88] Lim H Moon A. Inflammatory fibroblasts in cancer. Arch Pharm Res (2016) 39(8):1021–31. doi: 10.1007/s12272-016-0787-8 27384063

[89] Giannoni E Bianchini F Masieri L Serni S Torre E Calorini L. Reciprocal activation of prostate cancer cells and cancer-associated fibroblasts stimulates epithelial-mesenchymal transition and cancer stemness. Cancer Res (2010) 70(17):6945–56. doi: 10.1158/0008-5472.CAN-10-0785 20699369

[90] Spaeth EL Dembinski JL Sasser AK Watson K Klopp A Hall B. Mesenchymal stem cell transition to tumor-associated fibroblasts contributes to fibrovascular network expansion and tumor progression. PLoS One (2009) 4(4):e4992. doi: 10.1371/journal.pone.0004992 19352430 PMC2661372

[91] Krzysiek-Maczka G Targosz A Szczyrk U Strzalka M Sliwowski Z Brzozowski T. Role of helicobacter pylori infection in cancer-associated fibroblast-induced epithelial-mesenchymal transition in vitro. Helicobacter (2018) 23(6):e12538. doi: 10.1111/hel.12538 30246423 PMC6282800

[92] Yoshida K Murata M Yamaguchi T Matsuzaki K Okazaki K. Reversible human TGF-beta signal shifting between tumor suppression and fibro-carcinogenesis: Implications of smad phospho-isoforms for hepatic epithelial-mesenchymal transitions. J Clin Med (2016) 5(1):7. doi: 10.3390/jcm5010007 PMC4730132 26771649

[93] Nantajit D Lin D Li JJ. The network of epithelial-mesenchymal transition: potential new targets for tumor resistance. J Cancer Res Clin Oncol (2015) 141(10):1697–713. doi: 10.1007/s00432-014-1840-y PMC4382462 25270087

[94] Zhang J Thorikay M van der Zon G van Dinther M Ten Dijke P. Studying TGF-beta signaling and TGF-beta-induced epithelial-to-mesenchymal transition in breast cancer and normal cells. J Vis Exp (2020) 164:e61830. doi: 10.3791/61830 33191940

[95] Yoo JY Ku BJ Kim TH Il Ahn J Ahn JY Yang WS. Beta-catenin activates TGF-beta-induced epithelial-mesenchymal transition in adenomyosis. Exp Mol Med (2020) 52(10):1754–65. doi: 10.1038/s12276-020-00514-6 PMC8080580 33060769

[96] Zhang J Fan J Zeng X Nie M Luan J Wang Y. Hedgehog signaling in gastrointestinal carcinogenesis and the gastrointestinal tumor microenvironment. Acta Pharm Sin B (2021) 11(3):609–20. doi: 10.1016/j.apsb.2020.10.022 PMC7982428 33777671

[97] von Ahrens D Bhagat TD Nagrath D Maitra A Verma A. The role of stromal cancer-associated fibroblasts in pancreatic cancer. J Hematol Oncol (2017) 10(1):76. doi: 10.1186/s13045-017-0448-5 28351381 PMC5371211

[98] Fujii S Fujihara A Natori K Abe A Kuboki Y Higuchi Y. TEM1 expression in cancer-associated fibroblasts is correlated with a poor prognosis in patients with gastric cancer. Cancer Med (2015) 4(11):1667–78. doi: 10.1002/cam4.515 PMC4673993 26336878

[99] Zhai J Shen J Xie G Wu J He M Gao L. Cancer-associated fibroblasts-derived IL-8 mediates resistance to cisplatin in human gastric cancer. Cancer Lett (2019) 454:37–43. doi: 10.1016/j.canlet.2019.04.002 30978440

[100] Meyer C Sevko A Ramacher M Bazhin AV Falk CS Osen W. Chronic inflammation promotes myeloid-derived suppressor cell activation blocking antitumor immunity in transgenic mouse melanoma model. Proc Natl Acad Sci U S A (2011) 108(41):17111–6. doi: 10.1073/pnas.1108121108 PMC3193202 21969559

[101] Dorhoi A Du Plessis N. Monocytic myeloid-derived suppressor cells in chronic infections. Front Immunol (2017) 8:1895. doi: 10.3389/fimmu.2017.01895 29354120 PMC5758551

[102] Groth C Hu X Weber R Fleming V Altevogt P Utikal J. Immunosuppression mediated by myeloid-derived suppressor cells (MDSCs) during tumour progression. Br J Cancer (2019) 120(1):16–25. doi: 10.1038/s41416-018-0333-1 30413826 PMC6325125

[103] Gabrilovich DI Ostrand-Rosenberg S Bronte V. Coordinated regulation of myeloid cells by tumours. Nat Rev Immunol (2012) 12(4):253–68. doi: 10.1038/nri3175 PMC3587148 22437938

[104] Gabrilovich DI Bronte V Chen SH Colombo MP Ochoa A Ostrand-Rosenberg S. The terminology issue for myeloid-derived suppressor cells. Cancer Res (2007) 67(1):425; author reply 6. doi: 10.1158/0008-5472.CAN-06-3037 17210725 PMC1941787

[105] Lee CR Lee W Cho SK Park SG. Characterization of multiple cytokine combinations and TGF-beta on differentiation and functions of myeloid-derived suppressor cells. Int J Mol Sci (2018) 19(3):869. doi: 10.3390/ijms19030869 PMC5877730 29543758

[106] Ku AW Muhitch JB Powers CA Diehl M Kim M Fisher DT. Tumor-induced MDSC act via remote control to inhibit l-selectin-dependent adaptive immunity in lymph nodes. Elife. (2016) 5:e17375. doi: 10.7554/eLife.17375 27929373 PMC5199197

[107] Corzo CA Cotter MJ Cheng P Cheng F Kusmartsev S Sotomayor E. Mechanism regulating reactive oxygen species in tumor-induced myeloid-derived suppressor cells. J Immunol (2009) 182(9):5693–701. doi: 10.4049/jimmunol.0900092 PMC2833019 19380816

[108] Jayaraman P Parikh F Lopez-Rivera E Hailemichael Y Clark A Ma G. Tumor-expressed inducible nitric oxide synthase controls induction of functional myeloid-derived suppressor cells through modulation of vascular endothelial growth factor release. J Immunol (2012) 188(11):5365–76. doi: 10.4049/jimmunol.1103553 PMC3358566 22529296

[109] Rodriguez PC Ochoa AC. Arginine regulation by myeloid derived suppressor cells and tolerance in cancer: mechanisms and therapeutic perspectives. Immunol Rev (2008) 222:180–91. doi: 10.1111/j.1600-065X.2008.00608.x PMC3546504 18364002

[110] Lu C Redd PS Lee JR Savage N Liu K. The expression profiles and regulation of PD-L1 in tumor-induced myeloid-derived suppressor cells. Oncoimmunology. (2016) 5(12):e1247135. doi: 10.1080/2162402X.2016.1247135 28123883 PMC5214087

[111] Ghaleb AM Yang VW. Kruppel-like factor 4 (KLF4): What we currently know. Gene. (2017) 611:27–37. doi: 10.1016/j.gene.2017.02.025 28237823 PMC5391259

[112] Zhang J Zhu Z Wu H Yu Z Rong Z Luo Z. PODXL, negatively regulated by KLF4, promotes the EMT and metastasis and serves as a novel prognostic indicator of gastric cancer. Gastric Cancer (2019) 22(1):48–59. doi: 10.1007/s10120-018-0833-y 29748877 PMC6314994

[113] Ghaleb AM Nandan MO Chanchevalap S Dalton WB Hisamuddin IM Yang VW. Kruppel-like factors 4 and 5: the yin and yang regulators of cellular proliferation. Cell Res (2005) 15(2):92–6. doi: 10.1038/sj.cr.7290271 PMC1317089 15740636

[114] Wang L Chang EW Wong SC Ong SM Chong DQ Ling KL. Increased myeloid-derived suppressor cells in gastric cancer correlate with cancer stage and plasma S100A8/A9 proinflammatory proteins. J Immunol (2013) 190(2):794–804. doi: 10.4049/jimmunol.1202088 23248262

[115] Wang PF Song SY Wang TJ Ji WJ Li SW Liu N. Prognostic role of pretreatment circulating MDSCs in patients with solid malignancies: A meta-analysis of 40 studies. Oncoimmunology. (2018) 7(10):e1494113. doi: 10.1080/2162402X.2018.1494113 30288362 PMC6169582

[116] Lu X Horner JW Paul E Shang X Troncoso P Deng P. Effective combinatorial immunotherapy for castration-resistant prostate cancer. Nature. (2017) 543(7647):728–32. doi: 10.1038/nature21676 PMC5374023 28321130

[117] Highfill SL Cui Y Giles AJ Smith JP Zhang H Morse E. Disruption of CXCR2-mediated MDSC tumor trafficking enhances anti-PD1 efficacy. Sci Transl Med (2014) 6(237):237ra67. doi: 10.1126/scitranslmed.3007974 PMC6980372 24848257

[118] Wesolowski R Markowitz J Carson WE 3rd. Myeloid derived suppressor cells - a new therapeutic target in the treatment of cancer. J Immunother Cancer (2013) 1:10. doi: 10.1186/2051-1426-1-10 24829747 PMC4019895

[119] Aydin EM Demir TD Seymen N Said SS Oktem-Okullu S Tiftikci A. The crosstalk between h. pylori virulence factors and the PD1:PD-L1 immune checkpoint inhibitors in progression to gastric cancer. Immunol Lett (2021) 239:1–11. doi: 10.1016/j.imlet.2021.06.009 34363898

[120] Shen B Qian A Lao W Li W Chen X Zhang B. Relationship between helicobacter pylori and expression of programmed death-1 and its ligand in gastric intraepithelial neoplasia and early-stage gastric cancer. Cancer Manag Res (2019) 11:3909–19. doi: 10.2147/CMAR.S203035 PMC6525832 31190978

[121] Go DM Lee SH Lee SH Woo SH Kim K Kim K. Programmed death ligand 1-expressing classical dendritic cells MitigateHelicobacter-induced gastritis. Cell Mol Gastroenterol Hepatol (2021) 12(2):715–39. doi: 10.1016/j.jcmgh.2021.04.007 PMC8267570 33894424

[122] Lina TT Alzahrani S House J Yamaoka Y Sharpe AH Rampy BA. Helicobacter pylori cag pathogenicity island's role in B7-H1 induction and immune evasion. PLoS One (2015) 10(3):e0121841. doi: 10.1371/journal.pone.0121841 25807464 PMC4373751

[123] Beswick EJ Pinchuk IV Das S Powell DW Reyes VE. Expression of the programmed death ligand 1, B7-H1, on gastric epithelial cells after helicobacter pylori exposure promotes development of CD4+ CD25+ FoxP3+ regulatory T cells. Infect Immun (2007) 75(9):4334–41. doi: 10.1128/IAI.00553-07 PMC1951191 17562772

[124] Wu J Zhu X Guo X Yang Z Cai Q Gu D. Helicobacter urease suppresses cytotoxic CD8+ T-cell responses through activating Myh9-dependent induction of PD-L1. Int Immunol (2021) 33(9):491–504. doi: 10.1093/intimm/dxab044 34297096

[125] Li H Xia JQ Zhu FS Xi ZH Pan CY Gu LM. LPS promotes the expression of PD-L1 in gastric cancer cells through NF-kappaB activation. J Cell Biochem (2018) 119(12):9997–10004. doi: 10.1002/jcb.27329 30145830

[126] Han Y Liu D Li L. PD-1/PD-L1 pathway: current researches in cancer. Am J Cancer Res (2020) 10(3):727–42. PMC7136921 32266087

[127] Baumeister SH Freeman GJ Dranoff G Sharpe AH. Coinhibitory pathways in immunotherapy for cancer. Annu Rev Immunol (2016) 34:539–73. doi: 10.1146/annurev-immunol-032414-112049 26927206

[128] Ribas A Wolchok JD. Cancer immunotherapy using checkpoint blockade. Science. (2018) 359(6382):1350–5. doi: 10.1126/science.aar4060 PMC7391259 29567705

[129] Taube JM Anders RA Young GD Xu H Sharma R McMiller TL. Colocalization of inflammatory response with B7-h1 expression in human melanocytic lesions supports an adaptive resistance mechanism of immune escape. Sci Transl Med (2012) 4(127):127ra37. doi: 10.1126/scitranslmed.3003689 PMC3568523 22461641

[130] Dong P Xiong Y Yue J Hanley SJB Watari H. Tumor-intrinsic PD-L1 signaling in cancer initiation, development and treatment: Beyond immune evasion. Front Oncol (2018) 8:386. doi: 10.3389/fonc.2018.00386 30283733 PMC6156376

[131] Wang X Teng F Kong L Yu J. PD-L1 expression in human cancers and its association with clinical outcomes. Onco Targets Ther (2016) 9:5023–39. doi: 10.2147/OTT.S105862 PMC4990391 27574444

[132] Liu X Choi MG Kim K Kim KM Kim ST Park SH. High PD-L1 expression in gastric cancer (GC) patients and correlation with molecular features. Pathol Res Pract (2020) 216(4):152881. doi: 10.1016/j.prp.2020.152881 32089413

[133] Wu L Cai S Deng Y Zhang Z Zhou X Su Y. PD-1/PD-L1 enhanced cisplatin resistance in gastric cancer through PI3K/AKT mediated p-gp expression. Int Immunopharmacol (2021) 94:107443. doi: 10.1016/j.intimp.2021.107443 33581579

[134] Kawazoe A Shitara K Boku N Yoshikawa T Terashima M. Current status of immunotherapy for advanced gastric cancer. Jpn J Clin Oncol (2021) 51(1):20–7. doi: 10.1093/jjco/hyaa202 33241322

[135] Kim ST Cristescu R Bass AJ Kim KM Odegaard JI Kim K. Comprehensive molecular characterization of clinical responses to PD-1 inhibition in metastatic gastric cancer. Nat Med (2018) 24(9):1449–58. doi: 10.1038/s41591-018-0101-z 30013197

[136] Kono K Nakajima S Mimura K. Current status of immune checkpoint inhibitors for gastric cancer. Gastric Cancer (2020) 23(4):565–78. doi: 10.1007/s10120-020-01090-4 32468420

[137] Mohabati Mobarez A Soleimani N Esmaeili SA Farhangi B. Nanoparticle-based immunotherapy of breast cancer using recombinant helicobacter pylori proteins. Eur J Pharm Biopharm (2020) 155:69–76. doi: 10.1016/j.ejpb.2020.08.013 32798667

[138] Solbrig CM Saucier-Sawyer JK Cody V Saltzman WM Hanlon DJ. Polymer nanoparticles for immunotherapy from encapsulated tumor-associated antigens and whole tumor cells. Mol Pharm (2007) 4(1):47–57. doi: 10.1021/mp060107e 17217312

[139] Topalian SL Hodi FS Brahmer JR Gettinger SN Smith DC McDermott DF. Safety, activity, and immune correlates of anti-PD-1 antibody in cancer. N Engl J Med (2012) 366(26):2443–54. doi: 10.1056/NEJMoa1200690 PMC3544539 22658127

[140] Darvin P Toor SM Sasidharan Nair V Elkord E. Immune checkpoint inhibitors: recent progress and potential biomarkers. Exp Mol Med (2018) 50(12):1–11. doi: 10.1038/s12276-018-0191-1 PMC6292890 30546008

[141] Li B Chan HL Chen P. Immune checkpoint inhibitors: Basics and challenges. Curr Med Chem (2019) 26(17):3009–25. doi: 10.2174/0929867324666170804143706 28782469

[142] Taefehshokr S Parhizkar A Hayati S Mousapour M Mahmoudpour A Eleid L. Cancer immunotherapy: Challenges and limitations. Pathol Res Pract (2022) 229:153723. doi: 10.1016/j.prp.2021.153723 34952426

[143] Hegde PS Chen DS. Top 10 challenges in cancer immunotherapy. Immunity. (2020) 52(1):17–35. doi: 10.1016/j.immuni.2019.12.011 31940268

[144] Odunsi K. Immunotherapy in ovarian cancer. Ann Oncol (2017) 28(suppl_8):viii1–7. doi: 10.1093/annonc/mdx444 PMC5834124 29232467

[145] Rowshanravan B Halliday N Sansom DM. CTLA-4: a moving target in immunotherapy. Blood. (2018) 131(1):58–67. doi: 10.1182/blood-2017-06-741033 29118008 PMC6317697

[146] Keir ME Butte MJ Freeman GJ Sharpe AH. PD-1 and its ligands in tolerance and immunity. Annu Rev Immunol (2008) 26:677–704. doi: 10.1146/annurev.immunol.26.021607.090331 18173375 PMC10637733

[147] Dyck L Mills KHG. Immune checkpoints and their inhibition in cancer and infectious diseases. Eur J Immunol (2017) 47(5):765–79. doi: 10.1002/eji.201646875 28393361

[148] Kang YK Boku N Satoh T Ryu MH Chao Y Kato K. Nivolumab in patients with advanced gastric or gastro-oesophageal junction cancer refractory to, or intolerant of, at least two previous chemotherapy regimens (ONO-4538-12, ATTRACTION-2): a randomised, double-blind, placebo-controlled, phase 3 trial. Lancet. (2017) 390(10111):2461–71. doi: 10.1016/S0140-6736(17)31827-5 28993052

[149] Fuchs CS Doi T Jang RW Muro K Satoh T Machado M. Safety and efficacy of pembrolizumab monotherapy in patients with previously treated advanced gastric and gastroesophageal junction cancer: Phase 2 clinical KEYNOTE-059 trial. JAMA Oncol (2018) 4(5):e180013. doi: 10.1001/jamaoncol.2018.0013 29543932 PMC5885175

[150] Chakrabarti J Holokai L Syu L Steele NG Chang J Wang J. Hedgehog signaling induces PD-L1 expression and tumor cell proliferation in gastric cancer. Oncotarget. (2018) 9(100):37439–57. doi: 10.18632/oncotarget.26473 PMC6324774 30647844

[151] Xue LJ Su QS Yang JH Lin Y. Autoimmune responses induced by helicobacter pylori improve the prognosis of gastric carcinoma. Med Hypotheses (2008) 70(2):273–6. doi: 10.1016/j.mehy.2007.05.045 17681432

[152] Xue LJ Mao XB Liu XB Gao H Chen YN Dai TT. Activation of CD3(+) T cells by helicobacter pylori DNA vaccines in potential immunotherapy of gastric carcinoma. Cancer Biol Ther (2019) 20(6):866–76. doi: 10.1080/15384047.2019.1579957 PMC6605983 30786815

[153] Ramachandran M Jin C Yu D Eriksson F Essand M. Vector-encoded helicobacter pylori neutrophil-activating protein promotes maturation of dendritic cells with Th1 polarization and improved migration. J Immunol (2014) 193(5):2287–96. doi: 10.4049/jimmunol.1400339 25049358

[154] Peng X Zhang R Duan G Wang C Sun N Zhang L. Production and delivery of helicobacter pylori NapA in lactococcus lactis and its protective efficacy and immune modulatory activity. Sci Rep (2018) 8(1):6435. doi: 10.1038/s41598-018-24879-x 29691472 PMC5915382

[155] Deng Y Su W Zhu J Ji H Zhou X Geng J. Helicobacter pylori infection disturbs the tumor immune microenvironment and is associated with a discrepant prognosis in gastric de novo diffuse large b-cell lymphoma. J Immunother Cancer (2021) 9(10):e002947. doi: 10.1136/jitc-2021-002947 34645670 PMC8515460

[156] Wang T Liu X Ji Z Men Y Du M Ding C. Antitumor and immunomodulatory effects of recombinant fusion protein rMBP-NAP through TLR-2 dependent mechanism in tumor bearing mice. Int Immunopharmacol (2015) 29(2):876–83. doi: 10.1016/j.intimp.2015.08.027 26384537

[157] Ding C Li L Zhang Y Ji Z Zhang C Liang T. Toll-like receptor agonist rMBP-NAP enhances antitumor cytokines production and CTL activity of peripheral blood mononuclear cells from patients with lung cancer. Oncol Lett (2018) 16(4):4707–12. doi: 10.3892/ol.2018.9182 PMC6126164 30214604

[158] Wang T Du M Ji Z Ding C Wang C Men Y. Recombinant protein rMBP-NAP restricts tumor progression by triggering antitumor immunity in mouse metastatic lung cancer. Can J Physiol Pharmacol (2018) 96(2):113–9. doi: 10.1139/cjpp-2017-0186 28863272

[159] Codolo G Fassan M Munari F Volpe A Bassi P Rugge M. HP-NAP inhibits the growth of bladder cancer in mice by activating a cytotoxic Th1 response. Cancer Immunol Immunother (2012) 61(1):31–40. doi: 10.1007/s00262-011-1087-2 21833592 PMC11028894

[160] Hou M Wang X Lu J Guo X Ding C Liang T. TLR agonist rHP-NAP as an adjuvant of dendritic cell-based vaccine to enhance anti-melanoma response. Iran J Immunol (2020) 17(1):14–25. doi: 10.22034/iji.2020.80291 32224538

[161] Ramachandran M Yu D Wanders A Essand M Eriksson F. An infection-enhanced oncolytic adenovirus secreting h. pylori neutrophil-activating protein with therapeutic effects on neuroendocrine tumors. Mol Ther (2013) 21(11):2008–18. doi: 10.1038/mt.2013.153 PMC3831034 23817216

[162] Yang W Li Y Gao R Xiu Z Sun T. MHC class I dysfunction of glioma stem cells escapes from CTL-mediated immune response *via* activation of wnt/beta-catenin signaling pathway. Oncogene. (2020) 39(5):1098–111. doi: 10.1038/s41388-019-1045-6 31591480

[163] Panagioti E Kurokawa C Viker K Ammayappan A Anderson SK Sotiriou S. Immunostimulatory bacterial antigen-armed oncolytic measles virotherapy significantly increases the potency of anti-PD1 checkpoint therapy. J Clin Invest (2021) 131(13):e141614. doi: 10.1172/JCI141614 PMC8245183 34196308

[164] Ma J Jin C Cancer M Wang H Ramachandran M Yu D. Concurrent expression of HP-NAP enhances antitumor efficacy of oncolytic vaccinia virus but not for semliki forest virus. Mol Ther Oncolytics (2021) 21:356–66. doi: 10.1016/j.omto.2021.04.016 PMC8182386 34141872

[165] Ma J Ramachandran M Jin C Quijano-Rubio C Martikainen M Yu D. Characterization of virus-mediated immunogenic cancer cell death and the consequences for oncolytic virus-based immunotherapy of cancer. Cell Death Dis (2020) 11(1):48. doi: 10.1038/s41419-020-2236-3 31969562 PMC6976683

[166] Smyth EC Verheij M Allum W Cunningham D Cervantes A Arnold D. Gastric cancer: ESMO clinical practice guidelines for diagnosis, treatment and follow-up. Ann Oncol (2016) 27(suppl 5):v38–49. doi: 10.1093/annonc/mdw350 27664260

[167] Fashoyin-Aje L Donoghue M Chen H He K Veeraraghavan J Goldberg KB. FDA Approval summary: Pembrolizumab for recurrent locally advanced or metastatic gastric or gastroesophageal junction adenocarcinoma expressing PD-L1. Oncologist. (2019) 24(1):103–9. doi: 10.1634/theoncologist.2018-0221 PMC6324629 30120163

[168] Fuchs CS Doi T Jang RW-J Muro K Satoh T Machado M. KEYNOTE-059 cohort 1: Efficacy and safety of pembrolizumab (pembro) monotherapy in patients with previously treated advanced gastric cancer. J Clin Oncol (2017) 35(15_suppl):4003. doi: 10.1200/JCO.2017.35.15_suppl.4003 29040031

[169] Muro K Chung HC Shankaran V Geva R Catenacci D Gupta S. Pembrolizumab for patients with PD-L1-positive advanced gastric cancer (KEYNOTE-012): a multicentre, open-label, phase 1b trial. Lancet Oncol (2016) 17(6):717–26. doi: 10.1016/S1470-2045(16)00175-3 27157491

[170] Senchukova MA Tomchuk O Shurygina EI. Helicobacter pylori in gastric cancer: Features of infection and their correlations with long-term results of treatment. World J Gastroenterol (2021) 27(37):6290–305. doi: 10.3748/wjg.v27.i37.6290 PMC8515796 34712033

[171] Kayapinar AK Solakoglu D Bas K Oymaci E Isbilen B Calik B. Relationship of prognostic factors in stomach cancer with helicobacter pylori: A retrospective study. Acta Gastroenterol Belg (2021) 84(4):607–17. doi: 10.51821/84.4.012 34965043

[172] Tsai KF Liou JM Chen MJ Chen CC Kuo SH Lai IR. Distinct clinicopathological features and prognosis of helicobacter pylori negative gastric cancer. PLoS One (2017) 12(2):e0170942. doi: 10.1371/journal.pone.0170942 28152027 PMC5289528

[173] Tsuruta O Yokoyama H Fujii S. A new crystal lattice structure of helicobacter pylori neutrophil-activating protein (HP-NAP). Acta Crystallogr Sect F Struct Biol Cryst Commun (2012) 68(Pt 2):134–40. doi: 10.1107/S1744309111052675 PMC3274388 22297984

[174] Zanotti G Papinutto E Dundon W Battistutta R Seveso M Giudice G. Structure of the neutrophil-activating protein from helicobacter pylori. J Mol Biol (2002) 323(1):125–30. doi: 10.1016/S0022-2836(02)00879-3 12368104

[175] Kottakis F Papadopoulos G Pappa EV Cordopatis P Pentas S Choli-Papadopoulou T. Helicobacter pylori neutrophil-activating protein activates neutrophils by its c-terminal region even without dodecamer formation, which is a prerequisite for DNA protection–novel approaches against helicobacter pylori inflammation. FEBS J (2008) 275(2):302–17. doi: 10.1111/j.1742-4658.2007.06201.x 18076649

[176] Xiao Y Yu D. Tumor microenvironment as a therapeutic target in cancer. Pharmacol Ther (2021) 221:107753. doi: 10.1016/j.pharmthera.2020.107753 33259885 PMC8084948

[177] Kozlova N Grossman JE Iwanicki MP Muranen T. The interplay of the extracellular matrix and stromal cells as a drug target in stroma-rich cancers. Trends Pharmacol Sci (2020) 41(3):183–98. doi: 10.1016/j.tips.2020.01.001 PMC8812124 32014341

[178] Ridge SM Sullivan FJ Glynn SA. Mesenchymal stem cells: key players in cancer progression. Mol Cancer (2017) 16(1):31. doi: 10.1186/s12943-017-0597-8 28148268 PMC5286812

[179] Poh AR Ernst M. Targeting macrophages in cancer: From bench to bedside. Front Oncol (2018) 8:49. doi: 10.3389/fonc.2018.00049 29594035 PMC5858529

[180] Peranzoni E Lemoine J Vimeux L Feuillet V Barrin S Kantari-Mimoun C. Macrophages impede CD8 T cells from reaching tumor cells and limit the efficacy of anti-PD-1 treatment. Proc Natl Acad Sci U S A (2018) 115(17):E4041–E50. doi: 10.1073/pnas.1720948115 PMC5924916 29632196

[181] Li DK Wang W. Characteristics and clinical trial results of agonistic anti-CD40 antibodies in the treatment of malignancies. Oncol Lett (2020) 20(5):176. doi: 10.3892/ol.2020.12037 32934743 PMC7471753

[182] Sullivan RJ Hong DS Tolcher AW Patnaik A Shapiro G Chmielowski B. Initial results from first-in-human study of IPI-549, a tumor macrophage-targeting agent, combined with nivolumab in advanced solid tumors. J Clin Oncol (2018) 36(15):3013. doi: 10.1200/JCO.2018.36.15_suppl.3013

[183] Jia Z Zheng M Jiang J Cao D Wu Y Zhang Y. Positive h. pylori status predicts better prognosis of non-cardiac gastric cancer patients: results from cohort study and meta-analysis. BMC Cancer (2022) 22(1):155. doi: 10.1186/s12885-022-09222-y 35135494 PMC8822753

